# The Estimation of Acidic Behavior of Wood by Treatment with Aqueous Na_2_HPO_4_ Solution

**DOI:** 10.1155/2012/496305

**Published:** 2012-03-11

**Authors:** Güneş Uçar, Mualla Balaban Uçar

**Affiliations:** Faculty of Forestry of Istanbul University, Bahçeköy, 80895 Istanbul, Turkey

## Abstract

As a new approach, the acidity that wood exhibits under moderate conditions is assayed by stimulated dissociation of weak wood acids in lightly basic secondary phosphate solutions. To assure a sufficient dissociation of hardly soluble weak acids in the solution, the amount of wood suspended in Na_2_HPO_4_ solutions should be small but vary depending on the degree of acidity of wood species. However, the difficulties are associated with the titration of very dilute acids limiting the precision of the measurement. If the disintegrated wood is suspended in a secondary phosphate solution, the weak woods acids form the conjugate acid Na_2_HPO_4_ from secondary phosphate Na_2_HPO_4_ resulting in a pH fall of the solution. The decrease in the pH value in phosphate solution, which depends on the wood acidity, can be evaluated to estimate the acidity arising from wood under moderate conditions.

## 1. Introduction

It is well known that, except for very few species, the woods of trees exhibit an acidic behavior of weak-to-moderate degree. The source of this property is found in both, main components and extractives as well. Acetyl groups and uronic acid residues linked to the polyoses and some organic acids occurring in free but also ester forms are particularly responsible for the wood acidity of many species. Additionally, the contribution of polyphenolic substances such as tannins is also of great importance in some hardwoods, like the heartwoods of oak and chestnut, for their acidic property. The nature and variety of wood acids are extensively reviewed by Choon and Roffael [1].

The role of acidic behavior of wood in its mechanical and chemical utilization is well recognized long ago and several methods for estimation of wood acidity, for example, its pH value have been developed [2–10]. These methods generally use either water or dilute alkali (NaOH) and acid (HCl) solutions as extraction medium in which disintegrated wood is suspended mostly at room temperature but also at elevated temperatures.

Subramanian et al. [9] were first to choose a different medium for extraction of otherwise insoluble or hardly soluble wood acids. The use of sodium acetate solutions brought indeed more acids into solution and the authors have claimed being able to measure bonded acid groups conveniently and reliably. The sodium acetate method was also applied in some works on different wood species or to follow the change of wood acidity in some cases [11, 12].

The importance of wood acidity in its utilization demands the estimation of this property correctly and reliably. Used as massive wood, the natural wood acidity arising from free and soluble acids will be effective and the acidity of wood can easily be determined here by water extraction. However, during many technical and chemo-technical processes the heat and pressure and sometime the chemicals will cause splitting acetyl groups, esters or, sometimes, creating new acidic constituents as degradation products. As a new way to estimate the wood acidity which would make itself felt if the wood is exposed to not harsh but moderate conditions, we promoted the solubility and the dissociation of otherwise sparingly soluble and weak wood acids with Na_2_HPO_4_ solution at room temperature.

## 2. Analytical Aspects

There are several acidic wood components many of which are bound as esters, some exist in form of their salts but few are found in the free form. The common nature of all wood acids is that they are weak; that is, their acid exponents are generally <5. Thus, depending also on the amount of acid content the most woods can exhibit pH values usually higher than 4, in average 4.5–5 [13].

Though numerous weak acids or acidic groups are present in wood, the most acidic ones will govern the wood acidity. Designating them by the common formula HA we will observe following equilibria for the slightly soluble weak acids when the wood is suspended in water:

(1) $$\begin{array}{c} {\text{HA}}_{\left(\text{insoluble}\right)}^{\prime }⇌{\text{HA}}_{\left(\text{soluble}\right)}^{\prime \;}\\ {\text{HA}}_{\left(\text{soluble}\right)}^{\prime }+{\text{H}}_{2}\text{O}⇌{\text{A}}^{\prime -}+{{\text{H}}_{3}\text{O}}^{+}\\ \;{\text{HA}}_{\left(\text{insoluble}\right)}^{\prime \prime }⇌{\text{HA}}_{\left(\text{soluble}\right)}^{\prime \prime }\\ {\text{HA}}_{\left(\text{soluble}\right)}^{\prime \prime }+{\text{H}}_{2}\text{O}\;⇌\;{\text{A}}^{\prime \prime -}\;+\;{{\text{H}}_{3}\text{O}}^{+} \end{array}$$

and so on.

To simplify the things let us consider one weak acid HA_(insoluble)_ (we can also think it represents the average acidity and solubility of all):

(2) $$\begin{array}{c} {\text{HA}}_{\left(\text{insoluble}\right)}\;⇌\;{\text{HA}}_{\left(\text{soluble}\right)} \end{array}$$

(3) $$\begin{array}{c} {\text{HA}}_{\left(\text{soluble}\right)}\;+\;{\text{H}}_{2}\text{O}⇌{\text{A}}^{-}\;+\;{{\text{H}}_{3}\text{O}}^{+}. \end{array}$$

The first equation implies that the concentration of solubilized HA reaches a constant value at a given temperature since a surplus of wood is usually present. Like the saturated solution of a sparsely soluble salt, after filtering of wood from the suspension we obtain an aqueous solution which contains slightly soluble acids in their maximum concentrations. Insoluble wood acid esters too are subject to saponification in the solutions to more or less extent. There is always equilibrium between the concentration of esterified and saponified sides of a wood ester in solution. Thus, we can accept such an acid side produced from the saponification of an ester as it was the soluble side of an insoluble acid. Depending on the degree of their dissociation constants the acids are then more or less ionized. This means a weak acid may even fully ionize when its concentration is too low.

The solubility and ionization of a hardly soluble wood weak acid can be increased when a weak base is introduced in the solution, where it reacts with hydronium ions released from wood acid and forms a conjugate weak acid (of the weak base). With disodium hydrogen phosphate as the weak base, the reactions below would take place:

(4) $${\text{HA}}_{\text{soluble}}+{\text{H}}_{2}\text{O}\overset{K_{\text{HA}}}{⇌}{\text{A}}^{-}+{\text{H}}_{3}{\text{O}}^{+}$$

(5) $${\text{H}}_{3}{\text{O}}^{+}+{{\text{HPO}}_{4}}^{2-}\overset{K_{\text{b}}}{\underset{K_{\text{a}}}{⇌}}{\text{H}}_{2}{{\text{PO}}_{4}}^{-}+{\text{H}}_{2}\text{O}.$$

The weak wood acid HA is then ionized to limited extent and forms hydronium ions. While in pure water a simple equilibrium would occur, this equilibrium is disturbed by the second reaction which consumes hydronium ions. Thus, the equilibrium of first reaction will shift to the right and HA will be forced to dissociate more hydronium ions (principle of Le Châtelier). Since secondary phosphate is a weak base its reaction with hydronium is reversible too. Hence, there will be two equilibria in the system simultaneously (in the case of several wood acids present there will be a system with many equilibria). On the other side, when the solid wood is also in the same medium the solubility of HA will increase, in accord with the more extensive ionization of HA_(soluble)_:

(6) $${\text{HA}}_{\left(\text{insoluble}\right)}⇌{\text{HA}}_{\left(\text{soluble}\right)}.$$

Dissociation constants of HA and disodium phosphate (*K*_HA_, *K*_b_) determine the extent of ionization and neutralization reactions. The higher the both constants the bigger the conjugate acid dihydrogen phosphate formed. However, because the wood acidity will be determined later by titration of conjugate acid in aqueous medium this should have a dissociation constant large enough for being titratable with sufficient accuracy. This is one thing to be considered in seeking an appropriate base. Another thing is that the base should not be a source of an ion, which can also be dissociated by a wood acid itself. For instance, using sodium acetate in extraction medium we would have the acetate ions in surplus and the common-ion effect of these ions would stabilize the acetyl groups bound in wood polyoses. On the other hand the sodium acetate will promote the dissociation of other wood acids. The pronounced contribution of acetyl groups to the wood acidity in technique is emphasized by Roffael [7] and Choon and Roffael [1]. Since all reactions mentioned above are reversible they never allow the quantitative recovery of wood acids. In conclusion, the stimulating of the wood acid dissociation is just useful to estimate the capability of wood to release hydronium ions if it is exposed to mild-to-moderate conditions. But here, the contribution of acetyl groups to wood acidity is essential and should not be excluded. 

For the extraction medium we found the disodium phosphate as an appropriate weak base. Its dissociation constant is 1.6*·*10^−7^. In wood, the concentration of natural secondary phosphate ions is almost zero. The aqueous 0.1 M disodium phosphate solution has the pH value between 9.2 and 9.3.

## 3. Material and Methods

Chemicals of high purity (Merck) were used. 0.1 mol/L Na_2_HPO_4_ solutions were prepared by dissolving 71.628 g Na_2_HPO_4_ *·*12 H_2_O in 2.0 L of distilled and preboiled water in a volumetric flask. Freshly distilled water is sometimes supersaturated with carbon dioxide and it was boiled briefly to eliminate the gas. Distilled water which was preliminarily boiled and cooled down to the room temperature was always used to prepare either phosphate or standard acid and base solutions. Titrisol cartridges from Merck were used for preparing 0.1 mol/L standard hydrochloric solutions and these solutions again were taken to standardize 0.1 M sodium hydroxide solutions.

The wood specimens in form of discs were taken from the stems of black pine, alder, beech, spruce, and chestnut. Fresh wood was first chipped in a chopper machine and chips were air-dried in the shadow for about one month. After grinding and sieving, the 40 to 100 mesh fraction was used in the experiments.

The determination of wood acidity of each species was carried out in a series of eight to nine experiments where different weight of wood meal was suspended in 200.0 mL of 0.1 M disodium hydrogen phosphate for 24 hours. Based on the pH of the blank phosphate solution the lowest amount of wood was selected such that a pH drop of 0.5–0.6 was achieved, while this value for the greatest amount was kept between 1.0 and 1.1. The lowest and highest amounts are lying between one and ten g for most woods.

Titrations were performed in an automatic titrator (785 Titrino, Metrohm, with automatic temperature correction), the electrode of which is calibrated with the certificated buffers 7.00 and 9.00. Dynamic end-point titration method (DET, addition of variable volume increments to acquire about equal pH differences) was chosen with the equilibrium time of 30 seconds before the new reagent dispensing. The Metrohm software Tinet 2.4 enabled the evaluation of data. The desired portions of standard solutions up to 20.000 mL during some experiments were added with the dosimat (765 Dosimat, Metrohm). The phosphate solutions (100–200 mL) were measured with volumetric pipets.

## 4. Results and Discussion

### 4.1. Development of the Method and Preexperiments with Stearic Acid

From (4) and (5) it is apparent that the less the amount of slightly soluble weak acid the greater the portion dissolved and dissociated. Actually, all almost insoluble weak acids or other hardly soluble substances would become dissolved, when their concentration in water is sufficiently low. This would make it possible to extract them with an alkali and then to determine them titrimetrically. However, very dilute solutions of especially weak acids exhibit the big difficulty to recognize the correct end point of titration. Briefly, the titratable concentration of an acid depends on its dissociation constant; the higher this is the more dilute acid solutions can be handled.

To see the falsifying effect of the far going dilution in 0.1 M phosphate, the calculated mass of disodium phosphate dodecahydrate (7.1628 g) was transferred into 200.0 mL volumetric flasks to prepare 0.1 M phosphate solutions. A series of 0.1 M HCl standard solution (0.5 to 10.0 mL) was given to about 100 mL water portions which again were added to the disodium phosphate in volumetric flasks. The flasks were filled to specified volume with water and after inserting a stirring bar they were tightly stoppered, then, the solutions were stirred on a magnetic stirrer for 30 minutes.

Reacting strong hydrochloric acid with disodium phosphate should convert equivalent amount of weak base into its conjugate acid sodium dihydrogen phosphate. A 150.0 mL aliquot of each flask was titrated with standard alkali solution in titrator. A blank of 0.1 M phosphate solution was prepared and treated exactly in the same way, and its pH-value was measured under stirring. Since titrator acquires the pH value of solutions after 30-second equilibrium, the pH values of blank solution acquired for 1 minute after starting of stirrer were recorded. Stirring causes a pH fall in small extent (~0.05–0.09) occurring as a sharp drop at the very beginning (~0.09–0.11) followed by a slight rise. As the convention, the pH value of the blank was taken to mark the end point of titrations.

Though modern digital titrators with variable dynamic volume dispensing determine the inflection point(s) of a titration curve as end point(s), the Metrohm software Tinet 2.4 also enables to move along the curve and set these ones at desired pH values. In this way, the alkali consumption at a given pH value was read out for all titrations.  Table 1 gives the results of the dilution experiments.

From the results in Table 1 (second column) it is apparent that the titration of very dilute solutions (<0.001 mol/L HCl) leads the device to detect the inflection points with high alkali consumptions. Titration to the pH value of 0.1 M Na_2_HPO_4_ (9.20) delivers still higher results in this region. Because more insoluble weak acid would be recovered by dilution, it might be acceptable to take 2.5 mL HCl in 200 mL of phosphate as the lowest amount of acid (0.00125 mol/L; 5% higher yields). This amount of acid results in a pH drop of about 0.6 in the medium. 

**Figure 1:**
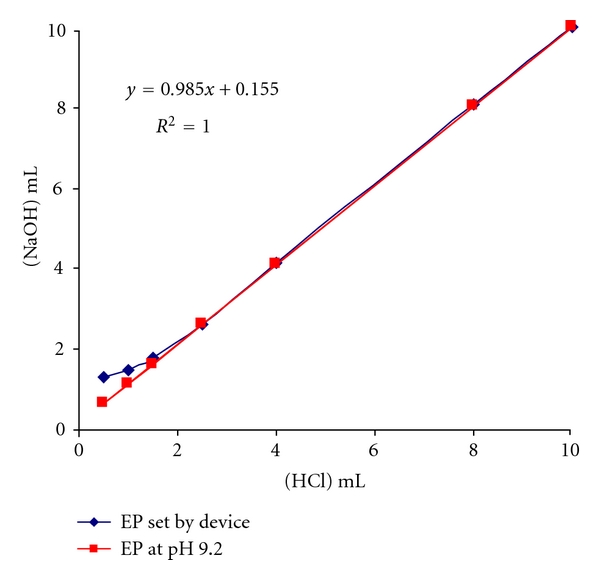
Graphical evaluation of alkali consumption of HCl-titration in Na_2_HPO_4_ solution.

The plots of alkali consumptions in second and fourth columns versus the HCl inputs (first column) result in the curves shown in Figure 1. 

The plot of consumption at the given pH value (9.2) results in a linear correlation with an intercept on ordinate axis. Since addition of 10 mL HCl gives the concentration in which the acid is being titrated quantitatively, a correction of alkali consumption can be performed in the way that the intercept value is subtracted from the consumed amount by decreasing it gradually according to the formula

(7) $$\;\begin{array}{cc} & \text{Alkali}\;\text{consumption}\\ & \;=\text{Alkali}\;\text{consump}.\;\text{at}\;\text{pH}\;9.2\\ & \;\;-\left(\frac{0.155}{9.995}\times \left(9.995-\text{Alkali}\;\text{consump}.\;\text{at}\;\text{pH}\;9.2\right)\right). \end{array}$$

The corrected alkali consumptions and the corresponding recoveries were depicted in the sixth and last columns of the Table 1, respectively. 

As the lowest allowable concentration has its limit so does the highest one too. Considering reversible reactions (4) and (5) both solubility and ionization of HA would be advanced by the excess of phosphate. Larger amount of wood would raise the absolute amount of insoluble HA and since we base the acidity on the wood weights, the relative yields would decrease. As it is demonstrated below with stearic acid an excess phosphate about at least 4-5 times is necessary to assure the conversion to higher extent. Based on the previous experiments with HCl above, where the dispensing of 10 mL 0.1 M HCl lowers the pH value down to 8.1, it is stressed in the experiments to weigh the wood meal of different species just in such highest amount which causes a pH drop of about 1–1.1. 

Stearic acid is a weak organic acid (MW = 284.49 g/mol) with very low solubility in water. It was used to test the ability of the weak bases sodium acetate and disodium phosphate to promote its dissociation. In order to estimate the acidity in the water or in a neutral electrolyte amounts between 0.2 to 1 g stearic acid were suspended in 200.0 mL of pure water or in 0.1 M NaCl solution in erlenmeyers with ground stopper. After 24 hours each suspension was filtered in a gooch crucible through fine porous ashless filter paper. Filtering is accomplished by applying light vacuum and 150.0 mL filtrate was taken for titration. The highest stearic acid recoveries in water and in NaCl solution were about 0.7 and 1%, respectively. A bit higher yield in NaCl solution is attributed to the well-known electrolyte effect in analytic chemistry [14]. 

A series of 7 weighing between 0.5 to 2.0 g of stearic acid was then suspended in 200 mL of 0.1 M sodium acetate solution in erlenmeyers which were treated in the same way. The results of titrations gave a nonlinear plot between mL consumption of alkali and the weight of acid, where the highest yield of ca. 5% is observed for the lowest weighing, while the yield decreased to about 1% in case of 2 g stearic acid. Since the highest weight (2 g), corresponding to about 7 mmol of stearic acid, was suspended in 20 mmol of acetate we thought in the beginning that this was the excess of sodium acetate enough during these experiments. However, because of the stearic acid is sparely soluble and since it is based on the whole stearic acid amount its insoluble part only lowered the yield. To determine the maximum acid recovery we decided to lower the amount of stearic acid down to one hundred milligrams in case of sodium acetate as extraction medium. 

**Figure 2:**
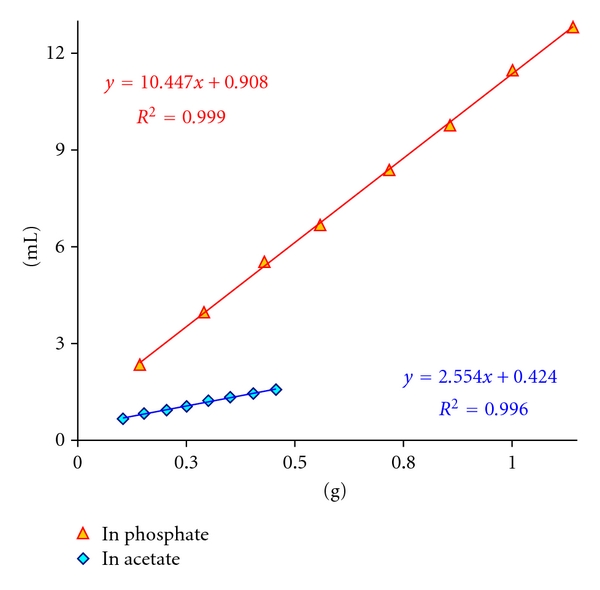
Titration of different amount of stearic acid in Na_2_HPO_4_ and CH_3_COONa solutions.

Taking secondary phosphate solution too, another series experiments was conducted in the same way described above. The related data of both experiments were summarized and visualized in Table 2 and Figure 2, respectively. 

The third and last columns (Table 2) display the stearic acid recoveries as mol percent. The graphical evaluation of the data in Figure 2 results again in *y*-intercepts, however, of greater values. The intercept leads to higher yields and the smaller is the alkali consumption the bigger is the relative error introduced this way. Like the titration of very dilute HCl solutions in 0.1 M phosphate, the intercept is a common problem and more pronounced with weak acids. We considered the intercept simply as an error in case of weak acids and subtracted it from the alkali consumption before calculating the yields. 

The stearic acid recovery in secondary phosphate solution is about 4 times bigger than that in acetate. In woods too, we obtained 3-4 times higher acidities with phosphate in comparison to the acetate (unpublished results). The stronger are the wood acids the higher will be the yield by the extraction with a weak base. The stearic acid should be considered as an example of a very weak acid with sparing solubility and its contribution to the wood acidity is then negligible. Even so, around one-third of stearic acid can be caught by the secondary phosphate as demonstrated above. 

### 4.2. The Determination of Wood Acidity with Disodium Hydrogen Phosphate Solution

The advanced ionization of stearic acid in secondary phosphate allowed for the development of a new method by using Na_2_HPO_4_ solutions to estimate the wood acidity when the wood is exposed to the moderate conditions. In this connection, the appropriate concentration of secondary phosphate solution and the time for reaching the equilibrium were searched and optimized first. 

**Figure 3:**
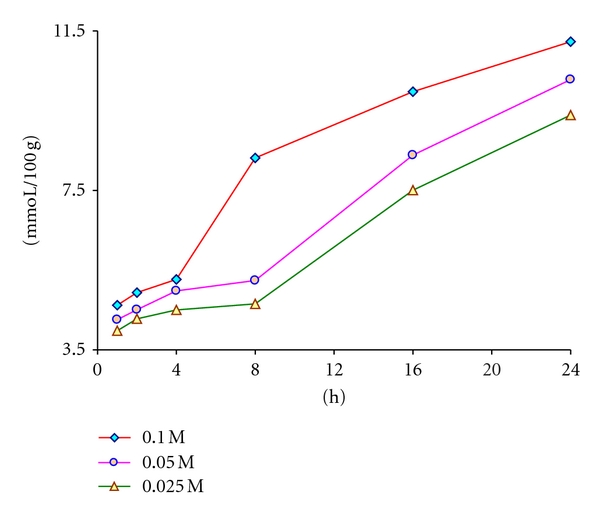
Effect of the time and concentration of Na_2_HPO_4_ solution on the extractability of wood acids.

At high ionic strengths, the interpretation of the behavior of solutions is difficult and therefore 0.1 M solution was selected as the highest concentration. Together with the 0.05 and 0.025 M Na_2_HPO_4_ solutions, three different concentrations were tested for the periods up to 24 hours (1, 2, 4, 8, 16, and 24 h). On absolute dry wood basis, 10.000 ± 0.005 g of beech wood were transferred into 300 mL erlenmeyer flask and thereafter 200.0 mL of 0.1 M phosphate solution were added. Giving a stirrer bar the flask was tightly closed with stopper and the suspension was stirred for one hour. In the same way, the experiments were repeated but for different periods and with 0.05 and 0.025 M phosphate solutions. Later it was observed that the stirring during the periods longer than eight hours has no effect at all. After elapsed time, each wood suspension is filtered and 150.0 mL of filtrate were titrated in device to the pH of blank phosphate solution (Figure 3). 

As expected from the reversible equations given in Section 2, both the elapsed time, during which the weak base is acting on the wood acids, and the concentration have impact on the extractability. In the first hours the extraction proceeds slowly and this can be ascribed to the low diffusion of the acids in compact wood structure. After a while the base causes a sufficient swelling which accelerates the reactions. The more concentrate 0.1 M phosphate has apparently this effect just after eight hours, whereas it takes longer with 0.05 and 0.025 M solutions. The statement should be made and underlined that the reversible reactions here make much longer periods necessary to achieve equilibrium. From Figure 3 it was then concluded that an extraction with 0.1 M Na_2_HPO_4_ solution for 24 hours would provide suitable conditions for the experiments. 

**Figure 4:**
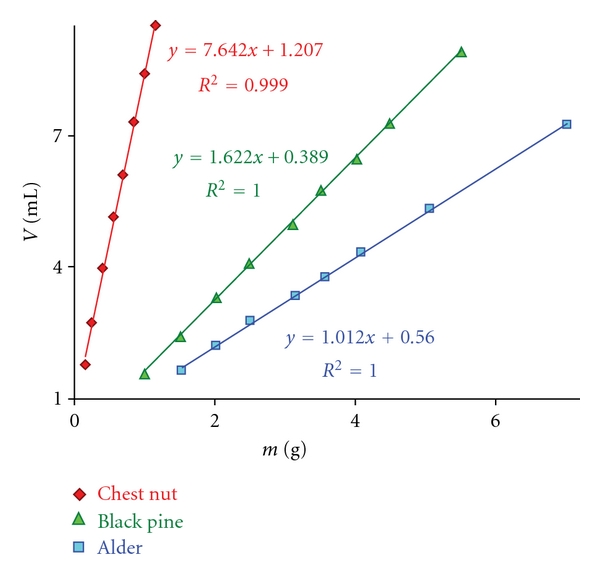
Graphical evaluation of consumed alkali of titration in dependence on sample weight.

Series of experiments including 8 to 9 determinations for each wood species were carried out to assay the related acidity. The results of three examples from soft- and hardwoods including one with extremely acidic wood (black pine, alder and chestnut heartwood) are given in Table 3.  Figure 4 also displays the plot of the volume of alkali (mL) consumed at the titrations against the weights of wood, resulting in linear correlations with very high *R^2^* values. 

The highest acidity found in the heartwood of chestnut originates particularly from tannins, while the resin acids in black pine are apparently responsible for the somewhat greater value. The acidities of the beech and spruce woods (not shown in the table) amounted to 11.5 and 12.0 mmol/100 g, respectively. Because of the very low resin acid content, the spruce exhibits a decrease in the wood acidity comparing to another softwood black pine. 

On the other side, the acetyl groups and 4-O-Me-glucuronic acid residues attached to the polyoses (xylans in hardwoods, mannans in softwoods) can be considered as the main source of wood acidity [13]. Their approximately known content can be compared with the data estimated by our phosphate method. The acetyl content in hardwoods amounts to about 4 percent [13, 15], that is, taking the mass of acetyl as reference (*M*_CH_3_CO_ = 43 g/mol), ca. 100 mmol of acetyl are present in 100 g hardwood. Based on a rough amount of 2 percent [13], similar calculation for 4-O-Me-glucuronic acid (MW = 191 g/mol) would result in ca. 10 mmol per 100 g of hardwood. Comparing to the hardwoods, the acetyl content of softwoods (galactoglucomannan) is smaller (≤1.5 percent [13, 15]). But the increased participation of Me-glucuronic acid residues in the arabinoxylan molecule of softwoods [13] which contain xylan in lower amount balances the uronic acid difference; thus, in softwoods the content of Me-glucuronic acid can also be approximated to 2 percent. According to these calculations and under the assumption that all acetyl groups and Me-glucuronic acid residues were cleaved off and reacted with secondary phosphate the wood acidity of hard- and softwoods should be estimated to be more than 100–110 mmol/100 g in case of first and 30–40 mmol/100 g in case of later ones. Much less acidity values found during the extraction with phosphate solution show, however, that the wood was only forced to exhibit its acidity to some extent. This promoted acidity corresponds then to a degree which would affect the utilization of wood in many practice areas that is, the production of plywood, particle- and fiberboard. 

**Figure 5:**
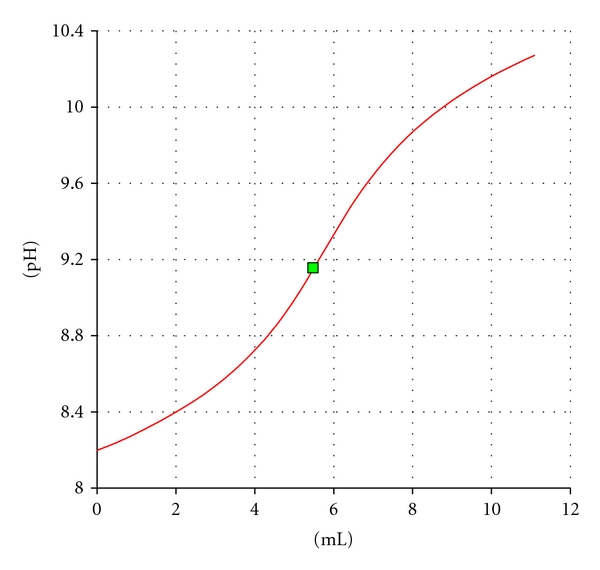
The typical titration curve of the Na_2_HPO_4_/NaH_2_PO_4_ solution obtained as filtrate from the wood suspension.

During the treatment with secondary phosphate, many weak acids in wood participate in reactions and at the end they are practically converted to one weak acid (NaH_2_PO_4_, conjugate acid of secondary phosphate). This is reflected by the titration curve where a modern and sensitive titrator itself can detect only one inflection point as the end point (Figure 5). 

**Figure 6:**
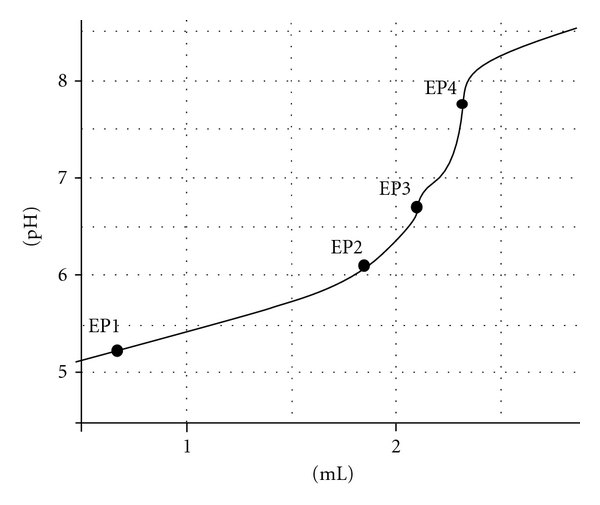
The titration curve of a hot water wood extract.

On the other side, the titration curves of the cold and hot water extracts as well as the extract from sodium acetate gave in average 3-4 end points indicating the presence of the acids of different strength (Figure 6). 

### 4.3. Suggested Methods to the Determination of Wood Acidity with Disodium Hydrogen Phosphate

From the results evaluated above and from our experiences with the phosphate solutions the following methods can be suggested for the determination of wood acidity that can be effective on the wood under moderate conditions in practice like production of plywood, particle- and fiberboard. 

(1) Method with a series of determinations: as explained and applied in this paper, 7 to 9 determinations should be preferred to assay the wood acidity. Therefore, the preparation of 2.0 liters 0.1 M Na_2_HPO_4_ solution is recommended as stock. (Use always distilled and briefly boiled water observing precautions that the water should not get excessive contact with air during cooling down and during experimenting otherwise.) For example, 9 determinations will use 1.8 liters of stock solution and about 100 mL blank are also kept in an erlenmeyer flask under same conditions. 300 mL erlenmeyer flasks with ground stopper should be used. First the wood is weighed in the flasks and they should be tightly closed soon after the 200.0 mL of phosphate solution is added. Two or three trials can be made before to estimate the pH drop caused by a given amount of wood. A series of weighing can then be made between the smallest and largest one, corresponding to a pH fall between ca. 0.6 and 1.1 from the pH of blank solution. Preparing vacuum flask, Gooch filter, and Filtering takes about 15 minutes, so it is advisable to put 20-minute intervals between determinations. The blank solution should be treated in the same way as the others and its pH value should be measured first under the same conditions the titrations made. This value serves the reference pH for end point of titrations. We observed a very small difference (0.01–0.02 decrease) in pH between untreated and filtered blank solutions. 

The flasks are then kept at 25 ± 2°C for 24 hours. The higher and lower temperatures affect the equilibrium resulting in some increase and decrease in the acidity. It is then recommended to keep the flasks in a water bath with a thermostat when the room temperature exceeds the limits. 

After the titrations with standard 0.1 M NaOH solution, the weights of wood are plotted against mL consumption of alkali (*y*-axis) to evaluate the data. A linear correlation with a *R^2^* greater than 0.998 is usually obtainable and here the *y*-intercept is determined. The intercept value is then deducted from mL alkali consumptions before calculating the yields. 

(2) A simpler way to determine the acidity is to prepare such 2-3 wood suspensions, each in 200 mL phosphate, where after 24 hours a decrease in pH about 1.2 from the pH-value of blank can be achieved. The mL alkali consumptions can then be taken and averaged for the calculation of wood acidity in good approximation to the first method. 

### 4.4. The Estimation of the Acidity as a Function of the pH of the Phosphate Solutions

In the presence of Na_2_HPO_4_, the reactions of wood acids generate the conjugate acid NaH_2_PO_4_, thus a buffer system occurs in the solution. On the other side the conjugate bases of wood acids are also present in the medium. The excess of phosphate buffer will mainly determine and stabilize the pH value of solution. Indeed, the phosphate solutions after the suspended wood was filtered off displayed a stable pH which can be measured as exactly as ±0.001 in the still state. This observation brought us to the idea, to determine the acidity by using the decrease in pH which would then correspond to a known amount of standard alkali solution that is equal to the promoted wood acidity. 

**Figure 7:**
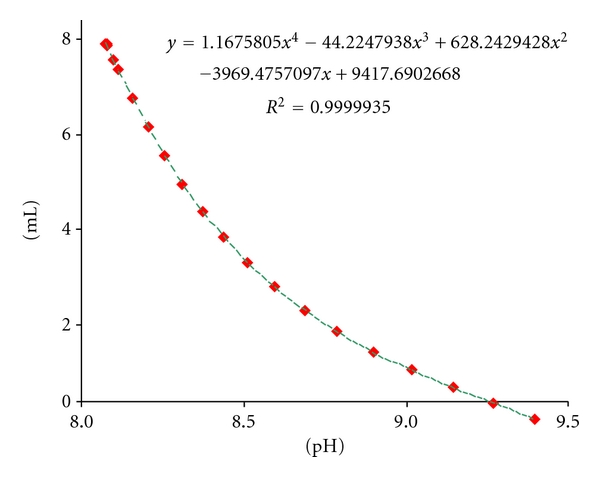
Graphical evaluation of the titration data of NaH_2_PO_4_/Na_2_HPO_4_-system.

7.1628 ± 0.0005 g Na_2_HPO_4_ *·*12 H_2_O were transferred in a 200 mL volumetric flask and dissolved about in 100 mL of water. After dispensing of 10.000 mL 0.1 M HCl (dosimat) the flask was filled to the mark with water. The solution in the flask was then stirred for about 30 minutes. 150.0 mL of the solution were titrated in the automatic titrator. The experiment was repeated twice. In graphical evaluation of the titration data, mL standard consumptions were subtracted from 7.500 mL to obtain the *y*-values. This enabled to calculate the wood acidity directly from the polynomial equitation of the curve depicted in Figure 7. 

The plot of pH versus calculated amount of mL (7.500-alkali consumption at the titration) results in a curve which is expressed by a polynomial equitation of the power of four. Achieving equitation with very high *R^2^* is of great importance, because a very good coincidence between the predicted data obtained by the formula and observed ones is ensured. At the calculations of mL alkali consumption for a given pH we used the numerical values of the coefficients in the correlation equitation by retaining 8 digits after decimal point. The pH value of the blank was taken again as the start point corresponding to zero mL alkali consumption. From this point on we can move upwards on the curve and calculate mL of alkali that corresponds to the equivalent acidity. The result is but valid for 150.0 mL and should be made for 200.0 mL by multiplying with the factor 4/3. 

**Figure 8:**
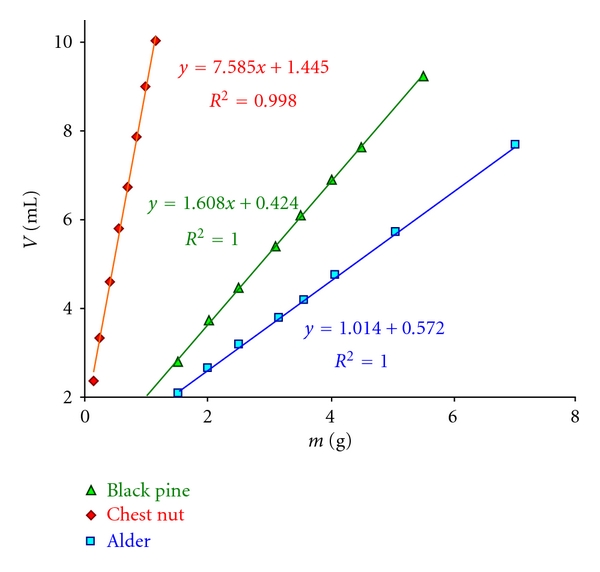
Graphical evaluation of calculated alkali consumption in dependence on sample weight.

Care should be exercised at the measurements of pH-value of wood filtrates. Here again the same conditions were applied, which were effective during the titration of Na_2_HPO_4_/NaH_2_PO_4_ solution with the device. Table 4 gives the results of calculations with the acidity yields based on the graphical evaluation (correction for intercepts according to the equations shown in Figure 8) for black pine, chestnut, and alder. 

In comparison to the titrations, smaller yields were obtained from the calculations of wood acidity as a function of pH. Somewhat bigger intercept values notable here are responsible for the slight decrease in yields but after all a good agreement between the predicted (calculations, Table 4) and observed (titrations, Table 3) yields is achieved. The calculated yields for the beech and spruce woods (not shown in the 4 and Figure 8) were 11.4 and 11.8 mmol/100 g, respectively. 

## Figures and Tables

**Table 1:** The effect of dilution on the titration of a strong acid in aqueous Na_2_HPO_4_ solution.

HCl	Alkali consumption mL						
added mL	End point set by device	% error	at pH 9.20	% error	Corrected*	% error	Recovery**(%)
0.50	1.3157	163.1	0.6333	26.7	0.4871	−2.4	97.6
1.00	1.4693	46.9	1.1427	14.3	1.0044	0.5	100.5
1.50	1.7713	18.1	1.6347	9.0	1.5041	0.3	100.3
2.50	2.6199	5.0	2.6240	4.8	2.5089	0.2	100.2
4.00	4.1572	3.9	4.1053	2.6	4.0134	0.3	100.3
8.00	8.0636	0.8	8.0400	0.5	8.0095	0.1	100.1
10.00	9.9916	−0.1	9.9947	−0.1	9.9947	−0.1	99,.9

*The data in the fourth column corrected according to (7) (see text).

**Based on corrected alkali consumption.

**Table 2:** Determination of stearic acid dissociated in CH_3_COONa and Na_2_HPO_4_ solution.

	Sodium acetate		Disodium hydrogen phosphate		
Alkali consumption at pH 7.8 (mL)	Stearic acid weight (g)	Yield* (%)	Alkali consumption at pH 9.2 (mL)	Stearic acid weight (g)	Yield* (%)
0.672	0.1040	6.78	2.344	0.1430	28.85
0.831	0.1526	7.58	3.967	0.2904	30.26
0.936	0.2044	7.13	5.536	0.4298	30.94
1.052	0.2505	7.13	6.673	0.5580	29.69
1.228	0.3008	7.60	8.376	0.7173	29.92
1.335	0.3513	7.37	9.764	0.8572	29.69
1.451	0.4045	7.22	11.467	1.0011	30.31
1.573	0.4565	7.16	12.801	1.1401	29.97

	Avg. Yield	7.25		Avg. Yield	29.95

*Based on standard alkali consumption corrected for intercept.

**Table 3:** Estimation of wood acidity by means of titration of wood-phosphate solutions.

	Black pine		Chestnut			Alder		
*V*/mL	*m_x_*/g	mmol/100 g*	*V*/mL	*m_x_*/g	mmol/100 g*	*V*/mL	*m_x_*/g	mmol/100 g*
1.959	1.0059	15.60^†^	2.191	0.1498	65.67^†^	2.053	1.5297	9.76
2.823	1.5016	16.21	3.121	0.2489	76.91	2.616	2.0236	10.16
3.716	2.0215	16.46	4.365	0.3994	79.08	3.161	2.5155	10.34
4.485	2.4981	16.39	5.531	0.5483	78.86	3.736	3.1660	10.03
5.383	3.1080	16.07	6.497	0.6974	75.86	4.176	3.5751	10.11
6.148	3.5011	16.45	7.693	0.8465	76.63	4.712	4.0781	10.18
6.871	4.0128	16.15	8.800	0.9912	76.60	5.707	5.0634	10.16
7.657	4.4847	16.20	9.897	1.1486	75.66	7.629	7.0145	10.08
9.296	5.5042	16.18						

	Avg:	16.26		Avg:	77.1		Avg:	10.10

*Based on alkali consumption corrected for intercept, ^†^not included to the average.

**Table 4:** Estimation of wood acidity as a function of pH-value of wood-phosphate solutions.

	Black pine			Chestnut				Alder			
pH	*V*/mL	*m_x_*/g	mmol/100 g	pH	*V*/mL	*m_x_*/g	mmol/100 g	pH	*V*/mL	*m_x_*/g	mmol/100 g
8.78	1.9820	1.0059	15.49^†^	8.715	2.3614	0.1498	61.18^†^	8.763	2.0772	1.5297	9.68^†^
8.647	2.8075	1.5016	15.87	8.576	3.3368	0.2489	76.01	8.673	2.6304	2.0236	10.05
8.529	3.7283	2.0215	16.35	8.437	4.6052	0.3994	79.13	8.598	3.1690	2.5155	10.15
8.449	4.4818	2.4981	16.24	8.334	5.7905	0.5483	79.26	8.525	3.7677	3.1660	9.95
8.365	5.4087	3.1080	16.04	8.264	6.7404	0.6974	75.93	8.481	4.1711	3.5751	9.93
8.309	6.1153	3.5011	16.26	8.192	7.8575	0.8465	75.75	8.424	4.7421	4.0781	10.03
8.254	6.8867	4.0128	16.10	8.127	9.0021	0.9912	76.24	8.340	5.7148	5.0634	9.98
8.206	7.6283	4.4847	16.06	8.075	10.020	1.1486	74.66	8.204	7.6607	7.0145	9.96
8.115	9.2286	5.5042	16.00								

		Avg:	16.11			Avg:	76.7			Avg:	10.1

^†^Not included into the average calculation.
